# Efficacy and Safety of Direct Factor Xa Inhibitors Versus Warfarin in Prevention of Primary and Secondary Ischemic Strokes in Non-Valvular Atrial Fibrillation: A Literature Review

**DOI:** 10.7759/cureus.9400

**Published:** 2020-07-26

**Authors:** Mina Emamy, Tehrim Zahid, Robert Ryad, Suhail M Saad-Omer, Nusrat Jahan

**Affiliations:** 1 Internal Medicine, California Institute of Behavioral Neurosciences and Psychology, Fairfield, USA; 2 Medicine, California Institute of Behavioral Neurosciences and Psychology, Fairfield, USA

**Keywords:** atrial fibrillation, direct factor xa inhibitors, warfarin

## Abstract

Ischemic stroke remains a major cause of mortality and morbidity in patients with atrial fibrillation. The use of appropriate anticoagulants reduces the risk of ischemic stroke in these patients. The current literature review is aimed to analyze the follow-up efficacy and safety of direct factor Xa inhibitors versus warfarin in the prevention of primary and secondary ischemic stroke, risk of major and minor bleedings, and food and drug interaction in patients with atrial fibrillation (AF). We selected PubMed as our database and we found 83,611 articles using the regular keyword 'atrial fibrillation'. We found 2,224 articles using the regular keywords 'direct factor Xa inhibitors' and 'atrial fibrillation'.

Finally, we obtained 326 studies using MeSH keywords: atrial fibrillation, direct factor Xa inhibitors, and warfarin. Ultimately, 46 articles were selected after applying the inclusion/exclusion criteria. All studies were randomized controlled trials (RCT) or clinical trials. Analysis of all studies showed that direct factor Xa inhibitors are superior to warfarin in the prevention of ischemic stroke in patients with non-valvular AF, with a lower rate of major and minor bleeding events and lower foods and drug interaction. Unlike warfarin, direct factor Xa inhibitors do not need frequent blood monitoring and dose adjustment. We found that warfarin and other vitamin K inhibitors may promote the calcification of heart valves and coronary arteries. There is some evidence that direct factor Xa inhibitors may slightly reverse these calcifications in coronary arteries and heart valves.

## Introduction and background

The number of patients with atrial fibrillation (AF) who need stroke prevention continues to rise. The prevalence of AF increases with age and is associated with a higher risk of ischemic stroke. The use of warfarin reduces the risk of ischemic stroke in patients with AF, but they need frequent monitoring and dose adjustment [1]. Ischemic stroke is considered as a focal neurological deficit from non-traumatic and non-hemorrhagic causes. AF is the cause of ischemic stroke in 15% of all ages and 30% of people over 80 years of age. The risk of ischemic stroke increases significantly with anticoagulant cessation [2].

The importance of a safe and effective prevention guideline with the best antiplatelets and anticoagulants combination is a major goal for medicine. Oral direct factor Xa inhibitors (xabans) are approved by the United States Food and Drug Administration (FDA) for the prevention of stroke. Warfarin is an antagonist of vitamin K. Xabans have a different effect in the clotting cascade. They act directly upon factor Xa. They have fewer drug and food interactions, and their location in the coagulation cascade promises their efficiency. There is no need to monitor their effects by checking the international normalized ratio (INR). This current review shows that xabans are at least as safe as warfarin in the elderly, patients with impaired liver and renal function, and in patients with a CHA2DS2-VASc score 2 or greater (scores that use factors like age, sex, history of stroke, hypertension and diabetes to estimate the risk of ischemic stroke in AF. A score of 2 or greater is moderate to high risk). Most physicians prefer these drugs over warfarin; however, there might be some limitations like patients’ kidney and liver function and the fact that they are not yet approved for valvular AF. Physicians need to consider the risk of bleeding, and the patient’s drug combination like their interaction with antiplatelet medications (like aspirin and clopidogrel).

There are some clinical benefits of xabans over warfarin. Based on current data, the best combination for the prevention of primary and secondary ischemic stroke in patients with AF would be aspirin plus clopidogrel and one xaban, such as apixaban, edoxaban, rivaroxaban, and darexaban [3]. There are still some challenging questions regarding the potential benefits of xabans over warfarin: How is their efficacy in the prevention of primary and secondary strokes compared to warfarin? How are their safety (minor and major bleedings) and food and drug interaction compared to warfarin? 

The presented literature review focused on the efficacy and safety of using xabans versus warfarin in the prevention of primary and secondary ischemic strokes in patients with non-valvular AF. This information will help clinicians to improve the outcomes of patients with AF.

## Review

Method and results

Data were collected manually on PubMed using parallel strategies derived from MeSH keywords and regular keywords. Table 1 represents all keywords used for this review.

**Table 1:** Data regarding the number of articles obtained using regular and MeSH keywords.

Regular and MeSH keywords	
Regular keyword: atrial fibrillation	
Total articles	83,611
Articles selected	1,095
Regular keyword: direct factor Xa inhibitors	
Total articles	2,333
Articles selected	132
MeSH keywords: atrial fibrillation, direct factor Xa inhibitors, warfarin	
Total articles	326
Articles selected	

This review has been generated after including the following inclusion/exclusion criteria. Table 2 represents the inclusion/exclusion criteria. 

**Table 2:** The inclusion/exclusion criteria.

Inclusion criteria	Exclusion criteria
Studies in the English language	Studies other than randomized clinical trials and clinical trials
Randomized controlled trials and clinical trials	Animal studies
Human studies	Studies that have been done more than 10 years ago
Studies in the last 10 years	Subjects of age below 18 years
Studies on patients with confirmed atrial fibrillation	
Subjects of age above 18 years	

We found 83,611 articles using the regular keyword 'atrial fibrillation'. We noted 2,224 articles using the regular keywords: 'direct factor Xa inhibitors' and 'atrial fibrillation'. Finally, we obtained 326 studies using MeSH keywords: 'atrial fibrillation', 'direct factor Xa inhibitors', and 'warfarin'. Ultimately, 46 articles were selected regarding the exclusion/inclusion criteria. All 46 studies that were chosen at the end had full articles available. They were all randomized controlled trials (RCTs) or clinical trials on human subjects. All articles have been in the English language since 10 years ago.

Study design 

All studies were clinical trials and RCTs. The maximum number of subjects among all reviewed articles was 21,105 and the minimum number of subjects was 45 [4,5]. Among all studies, 29 studies compared different xabans with warfarin. These alternative drugs included apixaban for six studies, rivaroxaban in fifteen studies, edoxaban for seven studies, and darexaban in one study [1-4,6-30]. Finally, six studies evaluated the effects of different types of non-vitamin K oral anticoagulants with warfarin [5,31-35]. This review shows that xabans are more efficient in the prevention of ischemic stroke in patients with non-valvular AF. There is less drug and food interaction when patients receive xabans. Also, the mortality rate, risk of major and minor bleedings, and hemorrhagic stroke are less common in patients who receive xabans compared to patients who receive warfarin.

**Figure 1:**
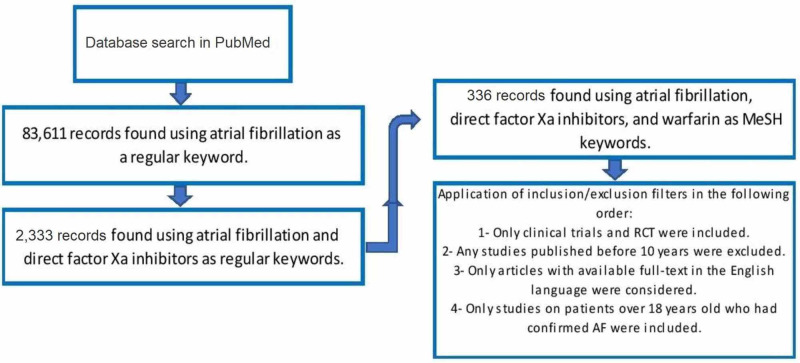
The flowchart that represents the selection process of the current literature review. AF, atrial fibrillation; RCT, randomized controlled trial

Figure 1 represents the flowchart with the selection process for this literature review. 

Discussion

AF is correlated with a higher risk of ischemic stroke. A stroke resulting from AF is more likely to be disabling than a non-AF stroke [28]. The importance of a more beneficial anticoagulant resulted in many clinical trials to compare these treatments. This review was performed to demonstrate that xabans are superior to warfarin in the prevention of ischemic stroke in patients with non-valvular AF. Also, we think that xabans have a lower rate of major and minor bleeding events and lower foods and drug interaction. We found that the majority of RCTs supported our hypothesis. We also found that xabans are at least as safe as warfarin in the elderly, patients with renal failure, patients with impaired liver function, and in patients with a CHA2DS2-VASc score 2 or greater. 

Major bleeding is explained by the International Society of Thrombosis and Haemostasis (ISTH) as a decline in the hemoglobin of at least two grams per deciliter, or if a patient needs at least two units of packed red blood cells, or if bleeding happens in critical sites, or bleeding that causes death. A non- major bleeding does not satisfy the criteria for major bleeding. Hematuria, epistaxis, gastrointestinal (GI) bleeding, and ecchymosis are the most frequent sites of non-major bleedings. A review of different studies demonstrated that the risk of minor and major bleedings for xabans is not more than warfarin [6]. Vitamin K antagonists like warfarin have a very narrow therapeutic index, many drug interactions, and also patients need to be monitored regularly [31]. There have been multiple studies that compared the outcome of patients with AF who are treated with xabans versus warfarin (Table 3).

In a RCT, 27 patients with cirrhosis received direct oral anticoagulants (DOAC) and 18 patients with cirrhosis received warfarin and low molecular weight heparin (LMWH). Only one patient in the DOAC group (4%) had major bleeding compared with five patients in the warfarin and LMWH groups (28%). The rate of thrombosis including ischemic stroke was 4% in the DOAC group vs. 6% in the warfarin and LMWH groups. These results support the preference of DOAC to warfarin and LMWH in patients with liver failure and cirrhosis [5]. Different clinical trials demonstrated that even in patients with a high-risk profile (elderly, patients with diabetes mellitus, cirrhosis, recurrent stroke, concomitant medications, and severe heart failure) still, xabans are superior to warfarin [27]. Vitamin K inhibitors inhibit post-translational activation of coagulation factors that are vitamin K dependents. On the other hand, they decrease the production of extrahepatic vitamin k-dependent proteins. This side effect promotes the calcification of heart valves and coronary arteries. Also, it has been shown that the consumption of vitamin K decreases the calcification of coronary arteries and heart valves [6].

Another randomized controlled study showed that apixaban not only has been superior to warfarin in the prevention of primary endpoints (ischemic stroke and other thromboembolic events), but it also prevents myocardial infarction (12%) [1]. An RCT compared warfarin with rivaroxaban in patients who use several concomitant medications. The study included the cohort of 5,101 patients with AF who used 0-4 medications, 7,298 patients who took 5-9 concomitant medications, and 1,865 patients who received 10 or more other medications. The concomitant medications were acetylsalicylic acid (ASA), angiotensin-converting enzyme inhibitors, beta-blockers, digitals, and diuretics. The study found out that the risk of stroke or peripheral embolism was not higher in patients who received fewer than 10 concomitant medications, but the risk of major bleeding events and all other causes of death increased by the number of concomitant medications. The risk of major bleeding was 11.64 per 100 patient-years for patients who took 0-4 medications, 14.79 per 100 patient-years for patients who received 5-9 medications, and 23.42 per 100 patient-years for patients who received 10 or more than 10 concomitant medications. In this study, there was not any significant different outcome between rivaroxaban and warfarin across all three groups [21].

However, two of the selected studies for this review had some different results. In one clinical trial, there was a significantly higher chance of GI bleeding in patients who received rivaroxaban compared to patients who were treated with warfarin (3.61 vs. 2.60 per 100 patient-years) [15]. Also, in another clinical trial, the efficacy of rivaroxaban vs. warfarin in the prevention of stroke was 1.71 vs. 1.95 per 100 patient-years in older patients (>75 years) [10]. The current literature review has some limitations: the study limits its analysis of studies in the previous 10 years on patients who are at least 18 years old, and RCTs and clinical trials who had a full article available. 

Table 3 summarizes some of the studies that compared the efficacy and safety of xabans with warfarin in patients with non-valvular AF.

**Table 3:** Summary of the studies that compared the efficacy and safety of direct factor Xa inhibitors with warfarin. AF, atrial fibrillation; ECG, electrocardiogram; HF, heart failure; ISTH, International Society of Thrombosis and Haemostasis; xaban, direct factor Xa inhibitor

Author/Date	Study Design	Subjects Received Xabans	Subjects Received Warfarin	Total Number of Subjects	Main Points
Giugliano et al. [4], 2013	Randomized controlled trial	7,035 (high dose) 7,034 (low dose)	7,036	21,105	Subjects in the warfarin group had 1.50% of developing an ischemic stroke or a systemic embolism (232 patients)., while only 182 participants from the high-dose edoxaban group (1.18%) and 253 participants from the low-dose edoxaban group (1.61% ) developed an ischemic stroke or an embolic event.
Granger et al. [1], 2011	Randomized controlled trial	9,120	9,081	18,201	All subjects that had two or more episodes of AF are documented by ECG and at least one risk factor for stroke. Apixaban decreased 60% in the risk of stroke or systemic embolism. Warfarin decreased 50% in the risk of stroke or systemic embolism. People who received apixaban had an ischemic stroke in 162 patients (0.97%), hemorrhagic stroke in 40 patients (0.24%), and major bleeding in 327 patients (2.13%). On the other hand, people who received warfarin had an ischemic stroke in 175 patients (1.05%.), hemorrhagic stroke in 78 patients (0.47%), and major bleeding in 462 patients (3.09%).
Bahit et al, [9], 2017	Randomized controlled trial	9,088	9,052	18,140	The study found that like major bleedings and non-major bleedings were also more common in patients treated with warfarin vs. apixaban (9.4 vs. 6.4 per 100 patient-years).
Patel et al. [17], 2011	Randomized controlled trial	7,131	7,133	14,264	Participants were at moderate to severe risk for stroke (ejection fraction less than 35%, high blood pressure, diabetes mellitus, or age >75 years). For people who received rivaroxaban (15-20 mg daily), fatal bleeding and intracranial hemorrhage in 82 patients (0.7%) vs. fatal bleeding and intracranial hemorrhage in 139 patients (1.2%) in the warfarin group.
Goodman et al. [20], 2014	Randomized controlled trial	7,111	7,125	14,236	Major bleeding events happened correspondingly in two groups (3.60 per 100 patient-years for rivaroxaban users vs. 3.45 per 100 patient-years for warfarin users). For both groups, the risk of bleeding increased by age, smoking, anemia, history of previous gastrointestinal bleeding, and prior usage of acetylsalicylic acid (ASA).
Bansilal et al. [10], 2015	Randomized controlled trial	7,131	7,133	14,264	In this study, 2,878 patients with diabetes and AF received rivaroxaban and 2,817 patients with diabetes and AF received warfarin. Also, 4,253 non-diabetic patients received rivaroxaban and 4,316 non-diabetic participants with AF received warfarin. Diabetic patients who received rivaroxaban had a lower rate of ischemic stroke compared to patients who received warfarin (1.48 vs. 1.55 per 100 patient-years). Non-diabetic patients had similar results (1.71 vs. 1.80 per 100 patient-years).
Magnani et al. [25], 2016	Randomized controlled trial	7,035	7,036	14,071	They evaluated 5,926 participants with no history of HF and 6,355 patients with class I-II HF, and 1,801 patients with class III-IV HF. The efficacy and safety of edoxaban in patients with mild to severe HF were similar (1.54 for no HF vs. 1.52 for mild HF, and 1.83 per 100 patient-years for severe HF). The result was similar to a patient without HF.
Fordyce et al. [14], 2016	Randomized controlled trial	6,359	6,253	12,612	Participants include 9,292 patients with normal renal function and 3,320 patients with renal failure (RF). As a result, patients with moderate RF (creatinine clearance of higher than 30 mL/min) do not need any dose adjustment for rivaroxaban (15-20 mg of rivaroxaban per day). In this study, patients with RF who received rivaroxaban had a slightly lower risk of ischemic stroke and systemic embolism compared with similar patients who received warfarin (1.54 vs.3.25 per 100 patient-years). There was not any difference in the chance of major bleeding between the two groups.
Lopes et al. [3], 2018	Randomized controlled trial	2,300	2,300	4,600	Apixaban was superior to vitamin K antagonists (warfarin) in the prevention of ischemic stroke. Also, patients who received apixaban with or without ASA have less possibility of ISTH major bleeding (31% reduction).
Hohnloser et al. [24], 2018	Randomized controlled trial	374	187	561	Patients who underwent catheter ablation have about 0.5% to 3% risk of thromboembolic effects. In this study, 374 patients who were supposed to have catheter ablation received 60 mg of edoxaban daily, and 187 patients were treated with warfarin (at least 21-28 days before the catheter ablation). The risk of an ischemic stroke for patients who received warfarin before and after the catheter ablation was less than 1% similar to the edoxaban group. The rate of major bleeding events was higher in patients who received warfarin (6.9%) compared with 1.6% in the edoxaban group.
Hong et al. [13], 2017	Randomized controlled trial	95	88	183	All patients in this study had at least one ischemic stroke before (confirmed by MRI). From 95 patients who were treated with rivaroxaban, 28 patients (29.5%) had a recurrent ischemic stroke, and 30 patients (31.6%) had an intracranial hemorrhage in four weeks. However, the number of patients in the warfarin group who experienced another ischemic stroke was 48 patients (54.5%) and 31 patients (35.6%) had an intracranial hemorrhage (in four weeks).
Zhu et al. [19], 2017	Randomized controlled trial	30	30	60	This study evaluated the metabolic benefits of rivaroxaban over warfarin in 60 patients who underwent a radiofrequency catheter ablation (RFCA). Metabolic indices like high-density lipoproteins (HDL), serum total proteins, globulin, and albumin were checked after RFCA. In 15 days following RFCA, the HDL level was 1.4 mmol/L for the rivaroxaban group and 1.1 mmol/L for the warfarin group. Also, the amounts of serum total protein (75.8 vs. 66.8 g/L), albumin (45.4 vs. 40.5 g/L), and globulin (30.4 vs. 26.3 g/L) were higher in the rivaroxaban group. It sounds that due to the different drug-food interactions of warfarin, rivaroxaban has some metabolic benefits over warfarin.

## Conclusions

This current literature review compared the primary efficacy (the prevention of ischemic stroke) and the incidence of bleeding events in patients who received xabans or warfarin. We aimed to demonstrate that xabans are superior or have at least the same efficacy of warfarin with a slightly less intracranial hemorrhage and major bleeding events. As xabans do not need continuous blood monitoring and less drug interaction, they are superior to warfarin in the prevention of a primary and secondary ischemic stroke in patients with non-valvular AF. Considering patients’ age, previous stroke, previous history of any major bleeding, CHA2DS2-VASc scores, and kidney and liver function, xabans have become the first choice of many physicians over warfarin for prevention of primary and secondary stroke in patients with non-valvular AF. Even though the risk of primary outcomes (ischemic stroke) and secondary outcome (any cause of death) increases by age, xabans are still superior to warfarin in the prevention of ischemic stroke events even in patients over 75 years of age. During our review, we found that like major bleedings, non-major bleedings were also more common in patients treated with warfarin.

There are still some challenging questions regarding the potential benefits of xabans over warfarin: Are they going to be approved for valvular AF? What are their other potential benefits over warfarin? Patients with valvular heart disease have more chances for AF, but we found very few studies that evaluated the advantages and disadvantages of xabans vs. warfarin in patients who had significant valvular heart disease and AF. Most studies on xabans excluded patients with moderate to severe mitral stenosis. Due to the limitation of current studies on subjects with valvular AF, the efficacy and safety of xabans vs. warfarin for patients with valvular AF can be tested in future studies.
